# Cannabidiol-Loaded Mucoadhesive PLGA Nanosphere-Chitosan Hydrogel Patch for Oral Therapeutic Applications

**DOI:** 10.3390/ijms27021127

**Published:** 2026-01-22

**Authors:** Badmaarag-Altai Chuluunbaatar, Jisu Park, Junyoung Song, Subin Mun, Ji-Hyun Kang, Kyung Hyun Min

**Affiliations:** 1 School of Pharmacy, Jeonbuk National University, Jeonju 54896, Republic of Korea; 2 Department of Bionanotechnology and Bioconvergence Engineering, Graduate School, Jeonbuk National University, Jeonju 54896, Republic of Korea; 3 Respiratory Drug Development Research Institute, Jeonbuk National University, Jeonju 54928, Republic of Korea; 4 Institute of New Drug Development, Jeonbuk National University, Jeonju 54928, Republic of Korea

**Keywords:** cannabidiol, PLGA nanosphere, mucoadhesive hydrogel, oral therapeutic, anti-inflammatory

## Abstract

Cannabidiol (CBD), the primary bioactive element of cannabis, has shown promise in alleviating pain and inflammation, although mechanisms in periodontal inflammation are not fully understood. To improve its limited solubility and mucosal permeability, the developed chitosan-based mucoadhesive hydrogel incorporating CBD-loaded PLGA nanospheres (CPN hydrogel) was characterized by FT-IR, SEM, particle size, rheological, swelling, and diffusion analyses, followed by biological evaluations, including wound-healing and RT-qPCR-based anti-inflammatory assays. The improved CPN hydrogel had a homogeneous shape, better viscoelastic behavior, and sustained drug release. Over 90% of CBD was released within 96 h, and Franz cell experiments showed improved permeability (124.1 μg/cm^2^ after 72 h). The gellan gum-based mucosal substrate significantly increased adhesion (1137.33 ± 142.25 s) compared to the control groups. Antioxidant studies indicated 73.65% DPPH radical scavenging, whereas antibacterial tests showed more than 99% suppression of *Staphylococcus aureus*. Furthermore, in vitro studies validated its wound healing and the downregulation of the inflammatory cytokines IL-6 and TNF-α. The results indicate that the CPN-loaded chitosan hydrogel has extended mucosal retention, strong antibacterial activity, and steady release of CBD. This underscores its significant potential as a targeted treatment for inflammatory oral diseases such as gingivitis and periodontitis.

## 1. Introduction

Periodontitis is a bacterial-induced chronic inflammatory disease that leads to the progressive destruction of the tooth-supporting tissues and alveolar bone due to plaque accumulation along the gingival margin [1,2]. Periodontitis is an infectious disease that influences various systemic risk factors and affects approximately 20–85% of the general population [1,3]. *Porphyromonas gingivalis* is a key pathogen in the diverse community of microbes that cause periodontal disease. It disrupts the balance of microbes in the mouth [4,5]. The pathogenicity largely stems from virulence factors, such as lipopolysaccharides (LPS), which play a critical role [6]. As the condition progresses, the supporting periodontal tissue degrades, ultimately leading to tooth mobility and, in extreme instances, tooth loss [7,8].

Cannabidiol (CBD), a non-psychoactive phytocannabinoid from Cannabis sativa with anti-inflammatory, immunomodulatory, and antimicrobial properties, is a promising bioactive compound for oral health applications [9,10,11]. In addition, CBD not only reduces inflammation, but also fights *P. gingivalis* very well [12]. A recent study demonstrated that CBD protects against periodontitis by suppressing inflammatory cytokines, preventing alveolar bone loss, and promoting periodontal tissue integrity [13]. Although CBD shows strong anti-inflammatory and antimicrobial potential, its clinical utility is limited by poor aqueous solubility (~12.6 µg/mL), high lipophilicity (logP ≈ 6.3), and low bioavailability (6–19%), making it imperative to develop advanced delivery systems to improve stability and mucosal retention [14,15,16]. PLGA nanoparticles, made from a biocompatible and biodegradable polymer [17] sanctioned by the U.S. Food and Drug Administration (FDA), function as an efficient carrier for hydrophobic CBD properties, help protect against early degradation, allow for longer drug release, and improve retention in inflamed periodontal tissues [18]. Nanoparticle systems, while advantageous, cannot independently address the swift elimination of drugs from the oral cavity due to salivary washout, enzymatic degradation, and mechanical forces. Consequently, a supplementary mucoadhesive matrix is required to maintain the nanoparticles at the application site and prolong their therapeutic efficacy [19]. Hydrogels have received significant attention for this application because of their viscoelastic properties, high water content, and capacity to conform to mucosal tissues closely. Polyvinyl alcohol (PVA) is a biocompatible hydrogel platform that improves nanoparticle retention in oral drug delivery applications [20,21].

**Figure 1:**
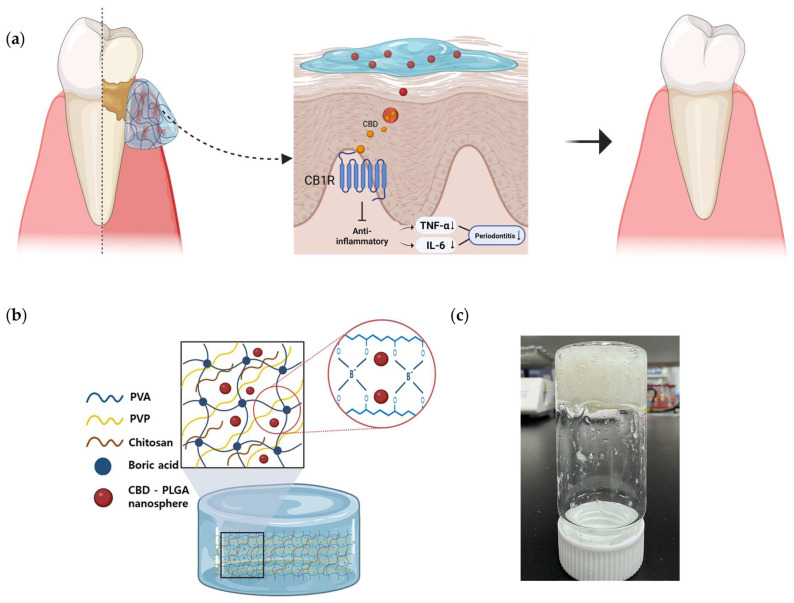
CPNloaded hydrogel system for periodontal treatment. (**a**) Illustration of CPN hydrogel application to inflamed periodontal tissue. (**b**) Schematic representation of the hydrogel network structure incorporating chitosan, boric acid, and CBD–PLGA nanospheres. (**c**) Experimental photograph showing the actual fabricated CPN hydrogel.

The CPN-loaded PVA–chitosan hydrogel creates a synergistic system in which PLGA nanoparticles enable prolonged CBD release, the mucoadhesive matrix improves mucosal retention, and chitosan has antibacterial properties against periodontal infections [22,23,24]. This dual-functional delivery method, which is enhanced by chitosan’s mucoadhesive and antibacterial properties [25], not only increases CBD’s physicochemical stability but also maximizes therapeutic exposure at the target location. This platform has the potential to decrease the frequency of doses, minimize systemic absorption, and enhance clinical outcomes in the treatment of periodontal inflammation. However, the oral therapeutic potential of CPN-loaded hydrogels remains unknown, and their physicochemical and biological properties have not been extensively assessed. As a result, the goal of this study was to create a chitosan-functionalized PVA-boric acid (PVA-BA) hydrogel integrating CPN to improve CBD stability, mucoadhesion, and local retention, thereby providing a viable method for targeted cannabis delivery in oral inflammatory therapy (Figure 1).

## 2. Results

### 2.1. Physical Properties of CBD-PLGA Nanosphere

**Figure 2:**
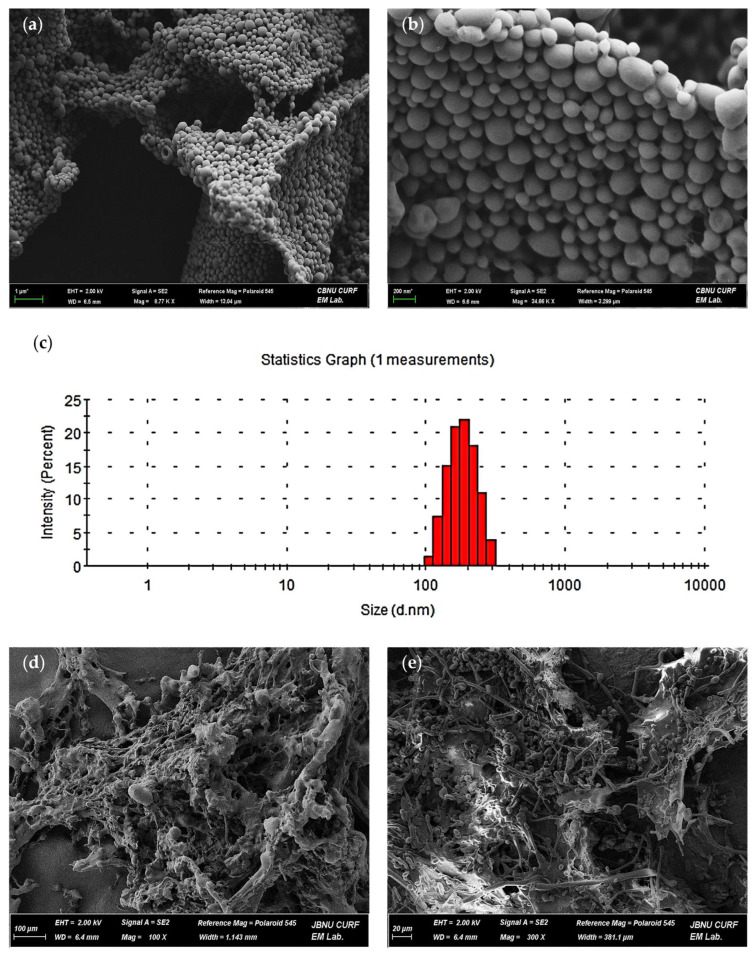
Morphological characterization of CBD-PLGA nanospheres and CPN hydrogel. SEM images of freeze-dried CBD-PLGA nanospheres showing (**a**) overall morphology and aggregation pattern (×8770) and (**b**) spherical particle shape with smooth surfaces (×34,660). (**c**) Particle size distribution of CBD-PLGA nanospheres showing a narrow. SEM images of the CPN hydrogel displaying (**d**) surface morphology and (**e**) cross-sectional porous network structure.

CPNs were prepared using a modified oil-in-water (O/W) emulsion solvent evaporation method, producing uniform colloidal dispersions after solvent removal. FE-SEM images (Figure 2a,b) revealed smooth, spherical nanospheres with uniform size and minimal aggregation, indicating successful encapsulation within the PLGA matrix. DLS analysis showed a hydrodynamic diameter (Z-average) of 178.0 ± 0.84 nm with a polydispersity index (PDI) of 0.09 ± 0.01 (Figure 2c), indicating a narrow size distribution and good colloidal uniformity. The zeta potential was −18.0 ± 0.5 mV, suggesting sufficient colloidal stability and a surface charge that may facilitate particle–matrix interactions. The loading content and encapsulation efficiency determined by HPLC were 3.61% and 24.17%, respectively (Table 1). These results confirm that the CPNs possess suitable physicochemical characteristics for incorporation into hydrogels designed for sustained mucosal drug delivery.

### 2.2. Characteristics of CPN Hydrogel Morphology

Field-emission scanning electron microscopy (FE-SEM) was used to examine the surface and internal structure of the CPN hydrogel at 100× magnification. The images depicted a porous, interconnected polymer network typical of a physically cross-linked matrix. The irregular, fibrous surface demonstrated potential for water absorption and drug transport (Figure 2d). The cross-sectional view (Figure 2e) exhibited a compact yet open internal structure, indicating uniform polymer distribution and efficient loading of CPN without observable aggregation or collapse. The microstructural features indicate that the CPN-incorporated hydrogel exhibits robust structural integrity and serves as an effective scaffold for targeted drug release.

### 2.3. FT-IR Spectroscopy of Cross-Linking and CPN Incorporation in the Hydrogel Matrix

**Figure 3:**
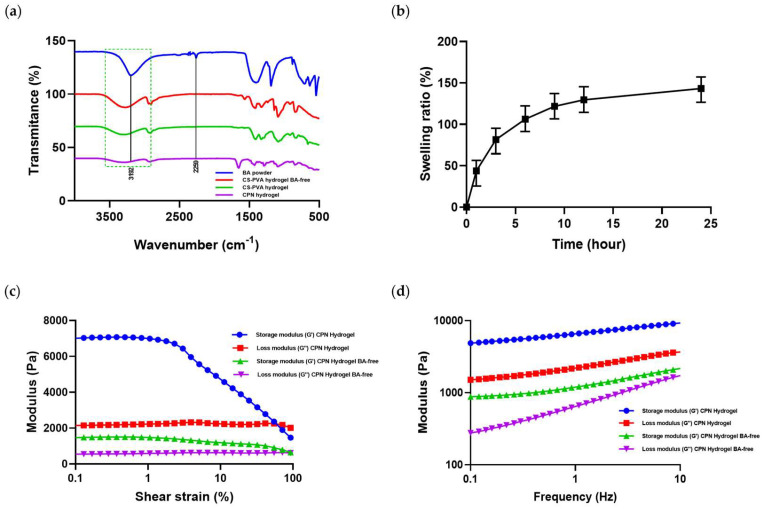
Physicochemical and rheological characterization of CPN hydrogels. (**a**) FT-IR spectra of boric acid, CS-PVA-BA free, CS-PVA, and CPN hydrogels showing peaks at 3192 and 2259 cm^−1^, confirming borate crosslinking and CBD encapsulation. (**b**) Swelling behavior of CPN hydrogels in PBS over 24 h, showing time-dependent water uptake. (**c**) Strain sweep and (**d**) frequency sweep profiles of CPN BA-free and CPN hydrogels, indicating storage (G′) and loss (G″) moduli. The data show the mean ± SD (n = 3).

Fourier-transform infrared (FT-IR) spectroscopy was used to examine chemical interactions and cross-linking in the hydrogel formulations (Figure 3a). Boric acid showed an O–H stretching band at 3192 cm^−1^, while pure PVA exhibited a broad band at 3200–3400 cm^−1^, indicating hydrogen bonding among hydroxyl groups. After boric acid incorporation (PVA–BA hydrogel), this band shifted to lower wavenumbers and broadened, confirming borate–diol complex formation. The CPN hydrogel showed further broadening and a characteristic peak at 2259 cm^−1^, corresponding to the C≡C stretch of CBD, confirming its molecular incorporation. Overall, the FT-IR results verified successful borate-mediated cross-linking and CPN integration within the hydrogel matrix.

### 2.4. Swelling Behavior of Hydrogel

The swelling behavior of the CPN hydrogel was evaluated in PBS (pH 7.4) (Figure 3b). The hydrogel showed rapid swelling during the first 4 h, reaching approximately 129% at 6 h, followed by a slower increase to near equilibrium (about 161% at 24 h). The borate-crosslinked network provided mechanical stability and prevented over-swelling, while the incorporation of CPN did not significantly affect the swelling ratio.

### 2.5. Rheological Behavior of Hydrogel

The viscoelastic behavior of the CPN hydrogels was characterized by amplitude (strain) and frequency sweep tests (Figure 3c,d). In the strain sweep range of 0.1–400%, the crosslinked CPN hydrogel exhibited a higher storage modulus (G′ ≈ 7000 Pa) than the non-crosslinked control (G′ ≈ 1500 Pa). The crosslinked sample maintained structural integrity at higher strain levels, while the control showed an earlier modulus crossover and rapid decline. In the frequency sweep (0.1–10 Hz), both G′ and G″ values of the crosslinked hydrogel were higher than those of the control, and G′ remained greater than G″ across all frequencies, indicating a predominantly elastic network.

### 2.6. In Vitro Release and Penetration Characteristics of CBD from CPN Hydrogel

**Figure 4:**
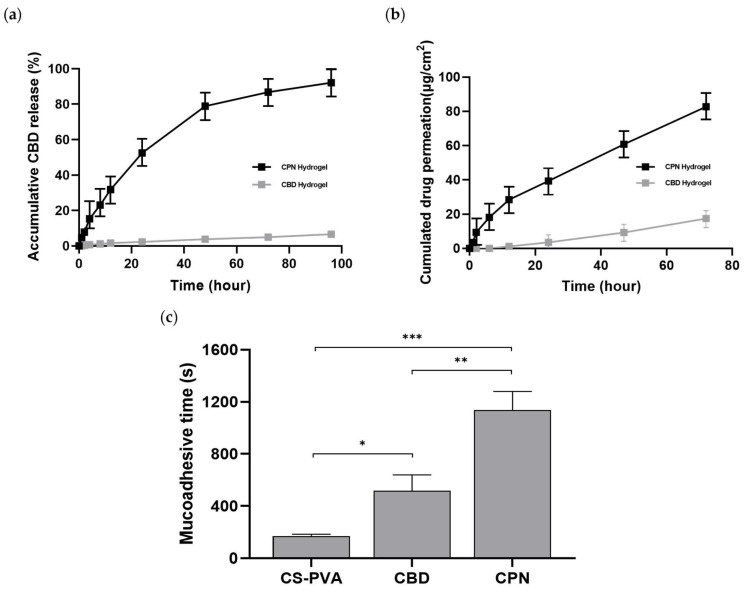
Functional characterization of CPN and non-encapsulated CBD hydrogels. (**a**) In vitro release profiles of CBD from CPN hydrogel and CBD in hydrogel (non-encapsulated) in PBS containing 1% Tween 80 over 96 h. (**b**) Cumulative permeation of CBD from CPN and CBD hydrogels across a synthetic membrane over 72 h using a Franz diffusion cell. (**c**) Mucoadhesive retention of CS-PVA, CBD-loaded, and CPN-loaded hydrogels on a gellan gum-based substrate. Statistical significance: * *p* < 0.05, ** *p* < 0.01, and *** *p* < 0.001. The data is shown as the mean ± SD (n = 3).

The diffusion of CBD from the hydrogel was assessed by in vitro release assays (Figure 4a). The CPN hydrogel released 7.8% of its CBD payload within the first hour, followed by 52.4% at 24 h and 92.1% at 96 h in a controlled manner. The controlled manner of CBD release from the CPN hydrogel is governed by a multi-stage mechanism, beginning with limited diffusion of surface-associated drug upon hydration, subsequent swelling-induced transport through enlarged pore channels, and gradual structural relaxation of PLGA nanospheres, which collectively suppresses burst release. In contrast, the non-encapsulated CBD exhibited a mere 6.6% release within the identical timeframe. This unexpectedly low release reflects the inherently poor aqueous solubility and limited diffusivity of free CBD, together with its tendency to adsorb onto experimental surfaces. By comparison, encapsulation within the PLGA-based hydrogel system maintains CBD in a finely dispersed state and provides continuous diffusion pathways during matrix hydration and polymer network relaxation, thereby enabling more efficient and sustained CBD release. Consistent with this concentration-dependent release behavior, the CBD release profiles from both the CPN hydrogel and the CBD hydrogel were well described by the first-order kinetic model, showing high correlation coefficients (r^2^ = 0.992 for CPN hydrogel and 0.986 for CBD hydrogel) (Table 2). This indicates that the release rate is governed by the remaining drug concentration within the matrix rather than being constant over time.

The permeation investigation (Figure 4b) demonstrated a significant disparity between the formulations. After 72 h, the CPN hydrogel released a cumulative total of 124.1 µg/cm^2^ across the membrane, while the control achieved just 17.5 µg/cm^2^. After the 12 h threshold, permeation escalated at a nearly uniform rate, resulting in a steady-state flow of 1.55 µg/cm^2^/h. The improved penetration results from the synergistic effects of the PLGA nanospheres and the hydrated PVA–BA network, which facilitate effective diffusion and transport over mucosal surfaces. The enhanced permeation is attributed to the complementary functions of the two components: the PLGA nanospheres stabilize CBD in a dispersed, highly available state, whereas the hydrated and mucoadhesive PVA–BA network prolongs residence time and provides interconnected diffusion pathways, collectively facilitating gradual transport across the mucosal surface. These findings underscore the CPN hydrogel as a resilient platform for the localized and sustained delivery of CBD displaying surface morphology and cross-sectional porous network structure.

### 2.7. Mucoadhesive Properties of Hydrogels

Figure 4c illustrates the comparative mucoadhesive performance of the hydrogels. The CS–PVA hydrogel exhibited detachment within approximately 3 min (170.33 ± 12.50 s), while the chitosan-free CPN hydrogel adhered for about 10 min (517.33 ± 122.09 s). In contrast, the complete CPN–chitosan formulation demonstrated firm adhesion for roughly 19 min (1137.33 ± 142.25 s), characterized by visible cohesive stretching that signified strong interfacial binding and structural integrity. The findings indicate that the integration of CPN and chitosan within the PVA–BA matrix significantly improves mucoadhesion, resulting in extended residence time and effectiveness for sustained mucosal drug delivery.

### 2.8. The Antibacterial Efficacy of CPN Hydrogel Against Staphylococcus aureus

**Figure 5:**
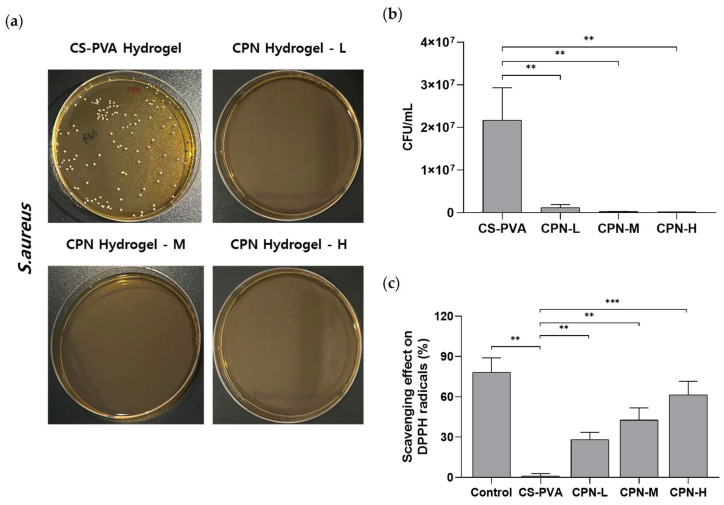
Antibacterial and antioxidant activities of CPN hydrogels. (**a**) Representative images of *Staphylococcus aureus* colonies on agar plates after treatment with CS-PVA hydrogel and CPN hydrogels loaded with 5, 10, and 20 mg of CPN. (**b**) Quantification of antibacterial activity as CFUs. (**c**) DPPH radical scavenging activity of free CBD, CS-PVA, and CPN hydrogels at various concentrations (5, 10, and 20 mg). ** *p* < 0.01, *** *p* < 0.001. The data is shown as mean ± SD (n = 3).

Antibacterial testing against *Staphylococcus aureus* was conducted via CFU analysis (Figure 5a,b). The CS-PVA hydrogel facilitated the formation of dense bacterial colonies, but hydrogels containing CPN at different doses (L-low dose 5 mg, M–medium dose 10 mg, H–high dose 20 mg of CPN) exhibited no bacterial growth. Quantification indicated that all CPN-containing groups attained over 99% suppression, with CFU levels nearing the assay’s detection threshold. The findings indicate that CPN hydrogels have significant antibacterial activity against *S. aureus*, rendering them intriguing for therapeutic applications where infection control is critical, such as wound management and mucosal treatments.

### 2.9. Antioxidant Activity of CPN Hydrogel

The antioxidant efficacy of CBD and CPN hydrogels was assessed utilizing the DPPH radical scavenging assay (Figure 5c). The scavenging action exhibited significant variation across the formulations based on CBD concentration. The free CBD group had considerable activity (50.83 ± 10.83%), while the CS–PVA hydrogel, devoid of CBD, displayed minimal activity (0.00 ± 9.17%), thus demonstrating that the antioxidant capacity was attributable to CBD. The CPN hydrogel–L (low-dose CPN 5 mg) exhibited an intermediate effect (27.30 ± 7.94%), whereas the full-strength CPN hydrogel–M (medium-dose CPN 10 mg) attained 57.10 ± 7.00%, and the CPN hydrogel–H (high-dose CPN 20 mg) formulation demonstrated the highest activity (73.65 ± 5.97%), signifying a distinct dose-dependent enhancement. The results demonstrate that encapsulating CBD within CPN enhances its antioxidant activity and provides increased stability in a concentration-dependent manner, highlighting its potential for addressing disorders related to oxidative stress.

### 2.10. Cytotoxic Effect of CPN Hydrogel

**Figure 6:**
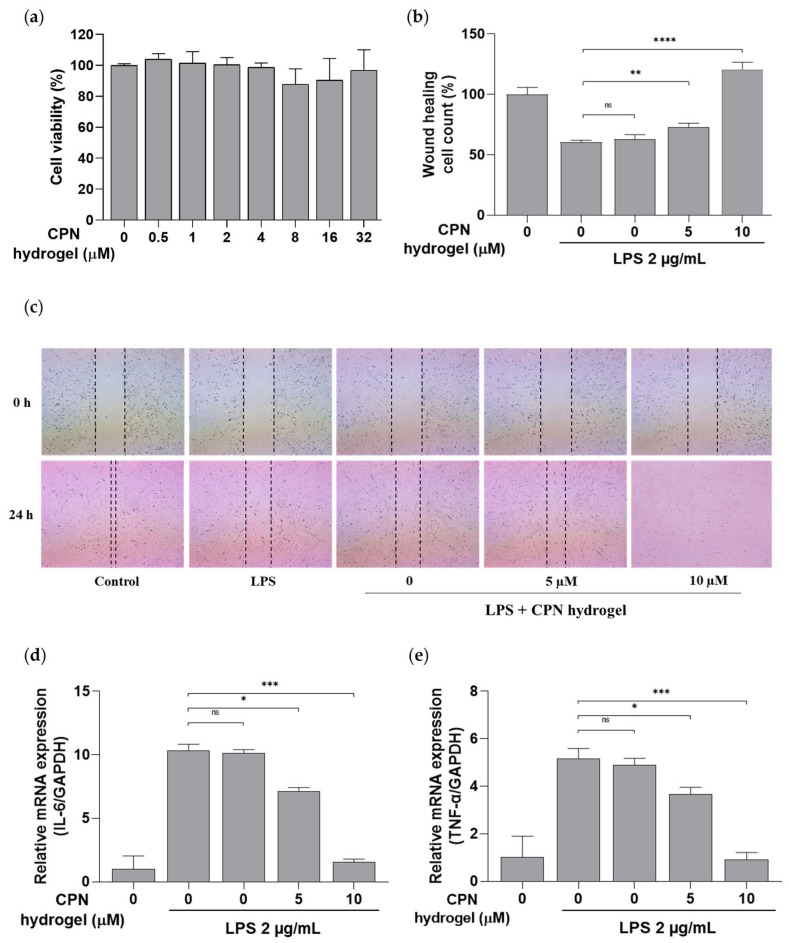
Anti-inflammatory, wound-healing, and cell viability effects of CPN hydrogel on HDF cells. (**a**) Cell viability of HDF cells after treatment with CPN hydrogel conditioned medium. For the wound-healing and RT-qPCR analyses, cells were stimulated with LPS (2 µg/mL) for 2 h, except for the control group, and then treated with CPN hydrogel conditioned medium for 18 h. (**b**) Quantification of wound closure in LPS-stimulated HDF cells treated with CPN hydrogel conditioned medium. (**c**) Representative images of the wound-healing (scratch) assay (4× magnification). All images were acquired at the same magnification. RT-qPCR analysis of pro-inflammatory cytokine mRNA expression levels of (**d**) IL-6 and (**e**) TNF-α under the same treatment conditions. Data are presented as means ± SD (n = 3). Statistical significance levels: * *p* < 0.05, ** *p* < 0.01, *** *p* < 0.001, and **** *p* < 0.0001; ns is non-significant compared to LPS and CS-PVA hydrogel groups.

The cytotoxic effects of the CPN hydrogels on HDF cells were evaluated by incubating with conditioned media containing various concentrations of CPN. As shown in Figure 6a, treatment with CPN hydrogel conditioned media did not significantly reduce cell viability compared with the untreated control. Notably, even at the highest CPN concentration, no pronounced decrease in cell viability was observed, suggesting that the gradual release of CBD from the CPN hydrogel matrix does not induce acute toxicity. These results support the safety profile of the CPN hydrogels and justify their further evaluation for periodontal applications.

### 2.11. In Vitro Evaluation of the Wound-Healing Effect of CPN Hydrogel

The in vitro wound-healing assay performed on HDF cells was conducted to evaluate the effects of CPN hydrogel and other treatments (Figure 6b,c). After LPS-induced inflammation, wound closure was assessed 18 h following treatment. As a result, treatment with media soaked in hydrogels containing 5 µM and 10 µM CPN significantly enhanced cell migration and wound closure compared with the CBD-free CS–PVA hydrogel, healthy, and other control groups; notably, the 10 µM formulation exhibited the most pronounced regenerative effect. These results indicate that CBD incorporation enhances therapeutic efficacy via sustained and biocompatible delivery.

### 2.12. Effect of CPN Hydrogel on Inflammatory Gene Expression Analysis

The RT-qPCR analysis was performed to evaluate the anti-inflammatory effects of the hydrogels by measuring changes in the mRNA expression of pro-inflammatory cytokine genes in HDF cells. All groups except the healthy control were stimulated with LPS to induce inflammation, resulting in markedly elevated cytokine expression of TNF-α and IL-6. However, following treatment with media soaked in hydrogels containing 5 µM and 10 µM CPN, a significant reduction in inflammatory gene expression was observed compared with the LPS-only and CBD-free CS–PVA hydrogel groups (Figure 6d,e). The 10 µM CPN hydrogel significantly decreased TNF-α and IL-6 expression, reaching values like those of the healthy control, which indicates a strong anti-inflammatory response.

## 3. Discussion

Periodontal diseases, such as gingivitis and periodontitis, represent prevalent chronic inflammatory conditions impacting the oral cavity. Gingivitis is a reversible inflammatory response confined to the gingival tissues, while periodontitis leads to irreversible destruction of the periodontal ligament and alveolar bone, ultimately jeopardizing tooth support and serving as a significant cause of tooth loss globally [26,27]. Traditional therapies exhibit restricted long-term efficacy. A significant limitation is the rapid elimination of topically applied medications caused by salivary flow and gingival crevicular fluid [28]. Moreover, prolonged antibiotic therapy raises issues regarding antimicrobial resistance and various systemic adverse effects [29,30]. Therefore, there is growing interest in creating localized, mucoadhesive drug delivery methods capable of extending drug retention inside periodontal tissues and improving overall therapeutic efficacy [31]. Consequently, there is an increasing focus on developing localized, mucoadhesive drug delivery systems that can enhance drug retention within periodontal tissues and improve therapeutic efficacy [32,33]. Our findings indicate that borate cross-linking generated an elastic and structurally stable hydrogel, an important feature for the constantly moist and dynamic oral environment. In contrast to conventional chitosan–PVA hydrogels that tend to erode rapidly and release drugs too quickly, the present system remained intact and enabled gradual CBD release for up to 96 h, thereby improving local retention and delivery. The strong agreement of the release profiles with the first-order kinetic model indicates that CBD release from both the CPN hydrogel and the CBD hydrogel is primarily governed by the remaining drug concentration within the matrix. As release proceeds and the internal CBD concentration decreases, the driving force for mass transfer is gradually reduced, resulting in sustained rather than abrupt drug release. This concentration-dependent behavior is characteristic of polymer-based delivery systems containing poorly water-soluble compounds, where drug transport is regulated by the availability of dissolved drug within the carrier rather than by a constant release rate.

CBD suppresses the expression of pro-inflammatory cytokines, including TNF-α and IL-6. Cytokines significantly contribute to chronic inflammation and tissue degradation in conditions like periodontitis, as evidenced by numerous studies [34,35]. Recent studies indicate that CBD exhibits antibacterial properties against significant periodontal pathogens, including *Staphylococcus aureus* and *Porphyromonas gingivalis* [36]. Researchers have increasingly examined innovative drug delivery platforms, including nanocarriers, liposomes, emulsions, solid dispersions, and hydrogels, to address the challenges related to CBD’s poor solubility, stability, and bioavailability [37]. Consistent with these efforts, our findings suggest that nanoparticle encapsulation preserves CBD functionality and prolongs cellular exposure, potentially mitigating pharmacokinetic constraints. Furthermore, compared with conventional antibiotic-loaded gels, the antibacterial effects achieved with this approach may reduce selective pressure for resistance while providing a multimodal antimicrobial effect.

Mucosal and transdermal delivery systems are particularly advantageous, as they circumvent hepatic first-pass metabolism and can be customized for localized, sustained release at specific sites [38,39]. Administering CBD directly to periodontal or mucosal tissues is particularly significant for oral inflammatory conditions, as it minimizes systemic exposure and enhances therapeutic outcomes [40,41]. In our study, the mucoadhesive strength of the CPN hydrogel increased by nearly sixfold compared with hydrogels lacking chitosan, indicating that the combination of chitosan and borate likely enhances interactions with mucin networks. Comparable improvements in adhesion have been reported for other mucoadhesive patches; however, most existing systems do not integrate such adhesion with nanoparticle-mediated sustained release. The present formulation provides both properties simultaneously, which may be particularly advantageous for localized periodontal therapy.

The CPN hydrogel was developed to overcome persistent limitations in CBD dispersion, and the data consistently indicate that this method significantly enhances CBD’s therapeutic efficacy in the oral environment. The technique successfully generated a structurally stable delivery platform by incorporating CBD into nanoscale PLGA carriers and embedding them within a chitosan-based hydrogel. Formulation stability is a critical requirement for the practical use of mucoadhesive hydrogels. In the present study, several findings collectively support the structural robustness of the CPN hydrogel. FT-IR analysis confirmed stable borate-mediated cross-linking, while FE-SEM images revealed a uniform porous network without particle aggregation or matrix collapse. Swelling experiments demonstrated controlled water uptake, indicating that the cross-linked structure prevents excessive expansion. Furthermore, rheological measurements showed a predominantly elastic profile with high storage modulus values, consistent with a mechanically stable network under deformation. Taken together, these observations indicate satisfactory short-term physical stability. Notably, the CPN hydrogel adhered effectively to moist oral surfaces and sustained CBD release over several days, suggesting an ability to withstand rapid salivary clearance, a significant limitation of conventional topical therapies. The formulation demonstrated significant biological advantages, including the suppression of key inflammatory pathways, inhibition of pathogenic microorganisms, and facilitation of cellular regeneration processes. The findings demonstrate that the CPN hydrogel effectively addresses key formulation challenges, enhancing its potential as a localized therapeutic approach. Despite these promising outcomes, several limitations must be acknowledged. Future studies incorporating animal periodontal models and clinically relevant simulations will be necessary to validate translational applicability and evaluate long-term safety and tissue integration.

## 4. Materials and Methods

### 4.1. Reagents

Poly (D, L-lactic-co-glycolic acid)-block-poly (glutamic acid) (PLGA quality level 100; Cat. No. 937754), polyvinyl alcohol (PVA ≥ 99% purity; Cat. No. 363146), polyvinylpyrrolidone (PVP quality level 100; Cat. No. PVP40), boric acid (BA ≥ 99.5% purity; Cat. No. B0394), gallic acid (97.5–102.5%; Cat. No. G7384), and chitosan (CS quality level 100; Cat. No. 448869) were purchased from Sigma-Aldrich (St. Louis, MO, USA). Formic acid (FA) was obtained from J.T. Baker (Phillipsburg, NJ, USA), and acetonitrile (ACN UN1648) was purchased from Thermo Chemical (Dreieich, Germany). Distilled water was purified using a Milli-Q^®^ system (Millipore, Burlington, MA, USA) and used throughout the study.

### 4.2. Formulation of CBD-PLGA Nanospheres

CBD-PLGA nanospheres (CPNs) were created by a modified oil-in-water (O/W) emulsion solvent-evaporation process. Briefly, 300 mg of PLGA was dissolved in 12 mL of dichloromethane (CH_2_Cl_2_) containing 40 mg of CBD, yielding a final drug concentration of 25 mg/mL. The organic component was carefully homogenized to achieve CBD dissolution and uniform dispersion inside the polymer matrix. A 2% (*w*/*v*) PVA solution was prepared separately by dissolving 480 mg PVA in 24 mL of distilled water, which served as the aqueous emulsifying phase. In a 100 mL glass beaker, the organic phase was gradually added to the PVA solution while magnetic stirring was kept constant. The mixture was then emulsified for 4 min with an ultrasonicator at 25% amplitude in pulse mode (30 s on/30 s off) to create a fine O/W emulsion. During sonication, the dispersion was maintained in an ice bath to keep the temperature below 10–15 °C, thereby preventing polymer degradation and CBD loss and ensuring a uniform O/W nanoemulsion. These ultrasonic conditions were selected because they consistently produced uniform nanospheres with a low polydispersity index while preventing overheating and minimizing polymer or drug degradation. Therefore, the same parameters were applied for all preparations in this study. To encourage nanosphere solidification and induce solvent evaporation, the initial emulsion was transferred to a 500 mL beaker containing 240 mL of distilled water and agitated at 300 rpm for 24 h at 40 °C. This phase expedited the gradual removal of dichloromethane, resulting in the formation of solid CPNs. The nanospheres were isolated using centrifugation at 12,000 rpm for 10 min, subsequently rinsed twice with distilled water to eliminate residual surfactant and unencapsulated CBD, and then stored at 4 °C. A laboratory lyophilizer was employed to freeze-dry the pure nanospheres for prolonged storage and subsequent hydrogel formation.

### 4.3. CBD-PLGA Nanosphere Characterization

The morphological characteristics of CPN were examined utilizing field-emission scanning electron microscopy (FE-SEM) and dynamic light scattering (DLS). The surface morphology, particle shape, and aggregation tendency were evaluated using a GeminiSEM 300 (Carl Zeiss, Jena, Germany). Lyophilized nanospheres were deposited on carbon-coated SEM stubs and subsequently sputter-coated with a thin coating of platinum under high vacuum to mitigate charge accumulation. Images were acquired at 3 kV, with morphological details recorded at several magnifications (200 nm and 1 µm scales). The hydrodynamic particle size, size distribution, and zeta potential were assessed using a Zetasizer Nano ZS (Malvern Instruments Ltd., Malvern, UK) employing non-invasive backscatter detection at 173°. Freeze-dried nanospheres were reconstituted in deionized water, briefly sonicated to ensure dispersion, and subsequently assessed at 25 °C. All measurements were conducted three times.

### 4.4. Determination of Drug Loading and Encapsulation Efficiency

The loading content and encapsulation efficiency of CPN were determined using high-performance liquid chromatography (HPLC). Analyses were performed on an UltiMate™ 3000 RS HPLC system (Thermo Fisher Scientific, Waltham, MA, USA) equipped with a Hypersil GOLD C18 column (4.6 × 250 mm, 5 µm). The mobile phase contained 0.1% formic acid in water (Solvent A) and 0.1% formic acid in acetonitrile (Solvent B), and isocratic elution was carried out at 1.0 mL/min. Detection was performed at 218 nm, and the column temperature was maintained at 40 °C. For CBD quantification, 2 mg of freeze-dried CPN were dissolved in 2 mL of acetonitrile, vortexed, and before injection, the sample was filtered using a 0.22 µm syringe filter. Samples were prepared in triplicate, and CBD concentrations were determined from a previously established calibration curve (regression equation: y = 0.2705x − 1.0662).

Drug loading (%) was calculated as the ratio of the amount of CBD measured in the nanospheres to the total weight of the nanospheres:

(1) $$\mathrm{Drug}\;\mathrm{Loading}\;(\%)\;=\;\left(\frac{Mass\;of\;CBD\;in\;nanospheres\left(mg\right)}{Total\;mass\;of\;nanospheres\left(mg\right)}\right)\times 100$$

The encapsulation efficiency (EE, %) was calculated as the ratio of the CBD amount recovered from the nanospheres to the initial amount of CBD used in the formulation process:

(2) $$\mathrm{Encapsulation}\;\mathrm{Efficiency}\;(\%)\;=\;\left(\frac{Mass\;of\;CBD\;in\;nanospheres\left(mg\right)}{Initial\;mass\;of\;CBD\;used\;in\;formulation\left(mg\right)}\right)\times 100$$

all analyses were performed under consistent chromatographic conditions, and calculations were based on the average of three independent measurements.

### 4.5. Preparation of Hydrogels

The hydrogels prepared according to Table 3 included CS-PVA hydrogel, CS-PVA hydrogel BA-free, CPN hydrogel, and CPN hydrogel BA-free. All formulations contained constant amounts of PVP, PVA, CS, and BA, whereas the CPN content was adjusted for specific experimental groups. A 15% (*w*/*v*) PVA solution was prepared by dissolving 750 mg of PVA in distilled water with continuous magnetic stirring at 400 rpm and heating at 90 °C for 1 h; the solution was subsequently cooled to room temperature. After cooling, 750 mg of PVP was added to the PVA solution and dissolved completely. For the CPN hydrogel formulations, 276.76 mg of CPN, equivalent to 10 mg of CBD based on a previously determined loading content (3.613%), was incorporated and dispersed uniformly. No CPN was added to the CS-PVA hydrogel formulations. After CPN incorporation, 20 mg of CS was added to each formulation to provide structural reinforcement and mucoadhesive properties. BA (53 mg) was incorporated into the CS-PVA hydrogel and CPN hydrogel formulations to induce physical cross-linking through interactions with PVA hydroxyl groups, whereas the CS-PVA and CPN BA-free hydrogel formulations were prepared without BA to serve as the non-crosslinked control. Each formulation was then adjusted to a final volume of 6 mL with distilled water, poured into pre-cleaned weighing dishes, and allowed to gel at room temperature under ambient conditions. All formulated hydrogels were sterilized under ultraviolet (UV) irradiation for 0.5 h before any biological assays.

### 4.6. Fourier Transform Infrared Spectroscopy (FT-IR) Spectroscopy

The chemical structure and intermolecular interactions of the hydrogels were analyzed using a Spectrum 3 FT-IR spectrometer (PerkinElmer, Shelton, CT, USA) equipped with a ZnSe attenuated total reflectance (ATR) accessory. Spectra were collected from 4000 to 500 cm^−1^ at a resolution of 4 cm^−1^, with 64 scans averaged per sample. Before measurement, the ATR crystal was thoroughly cleaned to avoid spectral contamination. Each hydrogel sample was placed directly onto the ATR surface and gently pressed to ensure uniform contact. Functional group vibrations associated with the polymer components (PVA, PVP) and cross-linking constituents (BA, CS) were recorded for structural characterization.

### 4.7. Morphology of the CPN Hydrogel

The surface morphology of freeze-dried hydrogels was studied using FE-SEM microscopy. The identical sample preparation and imaging settings as indicated in Section 4.3 were followed, except for using lower magnification ranges to view the overall hydrogel network architecture. Micrographs were obtained at 20–100 µm scales to analyze pore development and the gel matrix’s microstructure.

### 4.8. Rheology

The rheological behavior of the hydrogels was assessed using a rotational rheometer (Anton Paar MCR 92, Anton Paar GmbH, Graz, Austria) equipped with a cone-and-plate geometry (40 mm diameter, 1° cone angle, 0.1 mm gap). All measurements were performed at 25 °C using an integrated Peltier temperature-control system. To evaluate the viscoelastic properties, amplitude (strain) sweep tests were conducted over a strain range of 0.1–200% at a constant angular frequency. Storage modulus (G′) and loss modulus (G″) values were recorded as functions of applied strain, and the crossover point (G′ = G″) was identified to determine the limit of the linear viscoelastic (LVE) region.

### 4.9. Swelling Ratio Measurements

The swelling behavior of the hydrogels was evaluated by measuring their water uptake in phosphate-buffered saline (PBS, pH 7.4). Cylindrical hydrogel discs were dried in a vacuum desiccator at room temperature until a constant weight was obtained, and the dry weight (*W_d_*) was recorded using an analytical balance (0.1 mg sensitivity). Each dried disc was immersed in 10 mL of PBS and incubated at 37 ± 0.5 °C in sealed containers without agitation. Samples were removed at predetermined time points (1, 2, 4, 8, 12, and 24 h), gently blotted with lint-free filter paper to remove surface moisture, and immediately weighed to obtain the swollen weight (*W_t_*). All experiments were performed in triplicate (n = 3). The swelling ratio was calculated using *W_t_* and *W_d_*, and swelling profiles were plotted as a function of time.

The degree of swelling was calculated for each time point using the following equation:

(3) $$\mathrm{Swelling}\;\mathrm{ratio}\;=\;\left[\frac{\left(W_{t}-W_{d}\right)}{W_{d}}\;\right]\times 100$$

where *W_t_* is the weight of the hydrogel after swelling, and *W_d_* is the initial dry weight.

### 4.10. Mucoadhesive Retention Assessment

A time-based retention experiment on gellan-gum substrate assessed the hydrogels’ mucoadhesive qualities. To create consistent test surfaces, a 4% gellan gum solution with 2% glycerin was heated at 90 ± 5 °C and stirred continuously. It was then poured into 60 mm Petri plates and solidified at room temperature. This substrate composition was prepared according to the method described in [42], which demonstrated its suitability for assessing mucoadhesion. Simulated saliva (SS1) was prepared as reported by [43] and adjusted to pH 6.5. A small volume of SS1 was applied to the substrate before testing to maintain hydration at the hydrogel substrate interface. Mucoadhesion was assessed using a tilted-plate retention method. Each hydrogel sample (1.00 g) was placed at the center of the prehydrated gellan-gum surface and allowed to equilibrate for 30 s. The dish was then inclined to ~45°, and retention behavior was monitored over time. Tests were conducted in triplicate under identical conditions. Retention time was used as a comparative metric of mucoadhesive performance.

### 4.11. In Vitro Release Study

The in vitro release profile of CBD from CPN-loaded hydrogel and CBD in hydrogels was evaluated using a dynamic release method. Hydrogels containing the equivalent of 10 mg CBD were immersed in PBS (pH 7.4) containing 1% (*v*/*v*) Tween 80 and incubated at 37 °C with shaking at 150 rpm. At predetermined intervals (1, 2, 4, 8, 12, 24, 48, 72, 96, and 144 h), 40 mL of the release medium was discarded and immediately replaced with an equal volume of new pre-warmed medium to maintain sink conditions. For HPLC analysis, 0.7 mL of each sample was mixed with 0.7 mL acetonitrile, vortexed, and then centrifuged at 10,000 rpm for 10 min. The supernatant was analyzed for CBD content using the same HPLC system and chromatographic conditions described in Section 4.4. Cumulative release was calculated based on the initial CBD content of the hydrogel. The cumulative CBD release data were fitted to zero-order, first-order, Higuchi, Hixson–Crowell, and Korsmeyer–Peppas kinetic models to evaluate the release behavior. In these equations, *Q_t_* represents the cumulative amount of CBD released at time *t*, *Q*_0_ is the initial CBD content, *M_t_* and *M*_∞_ are the amounts of CBD released at time *t* and at infinite time, respectively, *k* is the corresponding release rate constant, and n is the diffusion exponent that describes the release mechanism. The goodness of fit was evaluated using the correlation coefficient (r^2^). The release kinetics models were analyzed using the following equations: Zero order (Equation (4)), first order (Equation (5)), Higuchi (Equation (6)), Hixson–Crowell (Equation (7)), Korsmeyer–Peppas model (Equation (8)).

(4) $$Q_{t}=k_{0}\;t$$

(5) $$log\;Q_{t}=log\;Q_{0}-(k_{1}t\;/\;2.303)$$

(6) $$Q_{t}=k_{H}t^{1/2}$$

(7) $${Q_{0}}^{1/3}-{Q_{t}}^{1/3}=k_{HC}\;t\;$$

(8) $$M_{t}{/M}_{\infty }=k_{KP}\;t^{n}$$

### 4.12. In Vitro Permeation Study

CBD permeation across a synthetic barrier was assessed using Franz diffusion cells (PermeGear, Hellertown, PA, USA). A Strat-M^®^ membrane was mounted between the donor and receptor chambers. The receptor compartment (12 mL) was filled with PBS (pH 6.8) containing 1% Tween 80, maintained at 37 ± 0.5 °C, and stirred at 600 rpm. Approximately 1 g of CPN hydrogel was placed in the donor compartment. At specified time intervals (1, 2, 4, 8, 12, 24, 48 h), 0.5 mL of the receptor medium was collected and immediately replaced with fresh pre-warmed receptor solution. Samples were mixed with acetonitrile (1:1, *v*/*v*), vortexed briefly, and centrifuged at 10,000 rpm for ten minutes. CBD content was determined using the same HPLC method as above. Permeation data were plotted as cumulative permeated amount versus time, and steady-state flux (Jss) was calculated.

### 4.13. Antibacterial Test

The antibacterial activity of the CPN hydrogels was evaluated against *Staphylococcus aureus* using a standard colony plate count assay. A bacterial suspension of *S. aureus* was prepared in sterile phosphate-buffered saline (PBS) at a concentration of 1 × 10^6^ CFU/mL. A 10 µL aliquot of the bacterial suspension was applied onto the surface of each hydrogel sample, followed by incubation at 37 °C for 2 h to allow direct contact between the bacteria and the hydrogel matrix. After incubation, each hydrogel was rinsed with 990 µL of sterile PBS to recover bacteria that adhered to or remained on the hydrogel surface. The recovered suspensions were serially diluted (1:10) by transferring 100 µL into 900 µL of sterile PBS at each dilution step. From appropriate dilutions, 100 µL was spread evenly onto LB agar plates. For the control group, 10 µL of the bacterial suspension was mixed with 990 µL of PBS and plated under identical conditions without hydrogel exposure. All plates were incubated at 37 °C for 24 h.

After incubation, colonies were counted on plates containing 30–300 colonies, and viable bacterial counts were expressed as CFU/mL using:

(9) $$\mathrm{Colony}\;\mathrm{Forming}\;\mathrm{Units}\;\mathrm{per}\;\mathrm{mL}\;(\mathrm{CFU}/\mathrm{mL})\;=\;\frac{Number\;of\;colonies}{Total\;Dilution\;Factor}$$

The antibacterial effect was calculated using:

(10) $$\mathrm{Antibacterial}\;\mathrm{ratio}\;(\%)\;=\;\left[\frac{\left(C_{control}-C_{sample}\right)}{C_{control}}\right]\times 100$$

where *C_control_* is the CFUs on control plates and *C_sample_* is the CFUs from CPN hydrogel-treated samples. All experiments were performed in triplicate (n = 3) under aseptic conditions.

### 4.14. Antioxidant Capacity of Hydrogels

The hydrogels’ antioxidant activity was assessed using the 2,2-diphenyl-1-picrylhydrazyl (DPPH) radical scavenging assay. A 0.1 mM DPPH solution was freshly prepared in absolute ethanol and stored in an amber container to prevent light-induced degradation. Hydrogel samples were weighed and dispersed in ethanol at concentrations corresponding to 5, 10, and 20 mg CPN loading. For each test, 1 mL of the hydrogel solution was mixed with 1 mL of DPPH solution, vortexed briefly, then incubated in the dark at room temperature for 30 min. After incubation, a UV-Vis spectrophotometer (GENESYS 150, Thermo Fisher Scientific, Waltham, MA, USA) was used to detect the absorbance at 517 nm. Ethanol served as the blank, and the DPPH solution without a sample was used as the negative control. Experiments were performed in triplicate (n = 3).

The formula for calculating the percentage of DPPH radical scavenging activity is as follows:

(11) $$\mathrm{Radical}\;\mathrm{scavenging}\;\mathrm{activity}\;(\%)\;=\;\left[\frac{\left(A_{control}-A_{sample}\right)}{A_{control}}\;\right]\times 100$$

where *A_control_* is the absorbance of the DPPH solution in the absence of any hydrogel sample, and *A_sample_* is the absorbance of the DPPH solution in the presence of CS–PVA hydrogel or CPN hydrogels at various concentrations.

### 4.15. Cell Culture and Conditioned Medium

Human Dermal Fibroblast (HDF) cells were cultured with Dulbecco’s modified Eagle’s medium (DMEM glucose-free, without sodium pyruvate) supplemented with penicillin (100 U/mL), streptomycin (100 µg/mL), and 10% fetal bovine serum (FBS) and incubated at 37 °C in a humidified 95% air and 5% CO_2_. To prepare the conditioned media, CPN hydrogels were weighed and incubated in growth medium at 37 °C in a shaker bath (100 rpm) for 24 h, resulting in conditioned media containing effective CPN concentrations of 5 or 10 µM. For comparison, CS-PVA hydrogels were weighed at an equivalent mass and incubated under the same conditions to obtain the control conditioned medium.

### 4.16. Cell Viability Assay

Cell viability was evaluated using hydrogel conditioned media to assess the potential cytotoxicity of CPN-loaded hydrogels. HDF cells were seeded in 96-well plates at a density of 1 × 10^4^ cells/well and allowed to attach overnight. The culture medium was then replaced with the hydrogel conditioned media and incubated for 24 h. After incubation, cell viability was assessed using the CCK8 assay kit (Dojindo Molecular Technologies, Kumamoto, Japan). Briefly, 10 µL of CCK8 solution was added to each well and incubated for 1–4 h at 37 °C. Absorbance was measured at 450 nm using a microplate reader.

### 4.17. Wound Healing Assay

In 6-well plates, HDF cells were grown at a density of 1 × 10^5^ cells/well until approximately 100% confluence. Using a sterile 200 µL pipette tip, a linear scratch was formed along the cell monolayer. Detached cells were then removed by washing twice with PBS. Except for the untreated control group, all cells were stimulated with LPS (2 µg/mL) for 2 h. The CPN hydrogel conditioned medium (5 and 10 µM) or CS-PVA hydrogel conditioned medium was subsequently treated for 18 h with LPS-stimulated HDF cells. A light microscope with a magnification of 4× was used to monitor wound closure and acquire photos at the specified time points.

### 4.18. RT-qPCR Analysis

HDF cells were cultured in 6-well plates and exposed to LPS (2 µg/mL) for 2 h. After stimulation, the cells were subsequently treated with CPN hydrogel conditioned medium (5 or 10 µM) or CS-PVA hydrogel conditioned medium for an additional 18 h. Total RNA was isolated from treated and control HDF cells using TRIzol reagent (Thermo Fisher Scientific, USA), as directed by the manufacturer. RNA purity and concentration were determined using a NanoDrop spectrophotometer (Thermo Fisher Scientific). One microgram of total RNA was reverse-transcribed into cDNA with a high-capacity cDNA synthesis kit. RT-qPCR was carried out using SYBR Green master mix (Enzynomics, Daejeon, Republic of Korea) on a QuantStudio real-time PCR system (Thermo Fisher Scientific). Gene expression levels of IL-6 and TNF-α were measured and normalized to glyceraldehyde 3-phosphate dehydrogenase (GAPDH). Relative expression was calculated using the 2^−ΔΔCt^ method [44]. All reactions were conducted in triplicate. The primer sequences used for amplification of pro-inflammatory pathway genes are listed in Table 4.

### 4.19. Statical Analysis

All experiments were performed in triplicate unless otherwise stated. Data are shown as means ± standard deviation (SD). Statistical analysis was conducted using the GraphPad Prism software program (version 8, GraphPad Software, San Diego, CA, USA). Comparisons between multiple groups were analyzed using one-way ANOVA and Tukey’s post hoc test. Statistical significance was defined as a *p*-value of <0.05. 

## 5. Conclusions

This study demonstrated that the hydrogels exhibited a uniform dispersion of nanospheres, stable borate crosslinking, and satisfactory swelling behavior, thereby maintaining structural integrity and adequate hydration. Additionally, strong mucoadhesion, sustained CBD release for up to 96 h, and enhanced transmucosal penetration facilitate the maintenance of therapeutic drug levels in inflamed oral sites. Biological investigations confirmed the therapeutic efficacy of the formulation. CPN therapy enhanced wound closure in HDF cells and reduced the expression of pro-inflammatory genes, such as TNF-α and IL-6, in the context of LPS-induced inflammation. The hydrogel’s regulated release and prolonged mucosal retention align with its anti-inflammatory and pro-healing properties. While antibacterial activity against *S. aureus* was observed, it constitutes an additional benefit. The CPN hydrogel serves as an effective localized therapeutic platform for oral inflammatory conditions, integrating substantial mucoadhesion, sustained CBD release, antioxidant properties, and anti-inflammatory signaling; however, further particular studies in animal periodontal models will be needed to confirm these findings under more physiologically relevant conditions.

## Figures and Tables

**Table 1:** CBD loading and encapsulation efficiency in PLGA nanospheres of CPN. Loading content (LC, % *w*/*w*) and encapsulation efficiency (EE, %) were determined for three independent CPN batches. The values are presented as mean ± SD (n = 3).

Formulation	LC (%, *w*/*w*)	EE (%)
CPN	3.613 ± 0.042	24.17 ± 0.31

**Table 2:** Kinetic parameters calculated.

Formulations	Zero Order		First Order		Higuchi		Hixson–Crowell		Korsmeyer–Peppas		
	r^2^	*k*_0_	r^2^	*k*_1_	r^2^	*k*_H_	r^2^	*k*_HC_	r^2^	n	*k*_KP_
CPN hydrogel	0.894	0.991	0.992	0.027	0.981	10.561	0.623	−0.033	0.985	0.670	5.380
CBD hydrogel	0.984	0.066	0.986	0.001	0.978	0.666	0.587	−0.014	0.980	0.778	0.199

**Table 3:** Composition of hydrogel formulations containing CBD-PLGA nanospheres. Each formulation contained fixed amounts of polyvinylpyrrolidone, polyvinyl alcohol, chitosan, and boric acid, with varying concentrations of CPN. All quantities are expressed in milligrams (mg) unless otherwise specified.

Samples	CBD (mg)	PLGA (mg)	PVP (mg)	PVA (mg)	CS (mg)	BA (mg)	DW (mL)
CS-PVA hydrogel	0.0	0.0	750.0	750.0	20.0	53.0	6.0
CS-PVA hydrogel BA-free	0.0	0.0	750.0	750.0	20.0	0.0	6.0
CPN hydrogel	10.0	266.8	750.0	750.0	20.0	53.0	6.0
CPN hydrogel BA-free	10.0	266.8	750.0	750.0	20.0	0.0	6.0

**Table 4:** RT-qPCR primer sequences.

Target Genes	Primer Sequence
GAPDH	Forward: 5′-GAAGGTGAAGGTCGGAGTC-3′ Reverse: 5′-GAAGATGGTGATGGGATTTC-3′
IL-6	Forward: 5′-CCACGGCCTTCCCTACTTC-3′ Reverse: 5′-TTGGGAGTGGTATCCTCTGTGA-3′
TNF-α	Forward: 5′-AGGGTCTGGGCCATAGAACT-3′ Reverse: 5′-CCACCACGCTCTTCTGTCTAC-3′

## Data Availability

The original contributions presented in this study are included in the article. Further inquiries can be directed to the corresponding authors.

## Abbreviations

The following abbreviations are used in this manuscript:

CBD	Cannabidiol
CPN hydrogel	CBD-PLGA nanosphere-loaded hydrogel
CPN	CBD-PLGA nanospheres
PLGA	Poly(D, L-lactic-co-glycolic acid)-block-poly(glutamic acid)
PVA	Polyvinyl alcohol
PVP	Polyvinylpyrrolidone
BA	Boric acid
CS	Chitosan
ACN	Acetonitrile
FA	Formic acid
FE-SEM	Field-emission scanning electron microscopy
DLS	Dynamic light scattering
SEM	Scanning electron microscopy
HPLC	High-performance liquid chromatography
FT-IR	Fourier-transform infrared spectroscopy
HDF	Human dermal fibroblast
DPPH	2,2-Diphenyl-1-picrylhydrazyl
LPS	Lipopolysaccharide
CFU	Colony Forming Units
IL-6	Interleukin-6
TNF-α	Tumor necrosis factor alpha
GAPDH	Glyceraldehyde 3-phosphate dehydrogenase
RT-qPCR	Reverse transcription quantitative polymerase chain reaction
